# Meat-eating justifications in Türkiye: cultural adaptation, validation, and correlates of the MEJ and 4Ns scales

**DOI:** 10.1186/s40359-025-03490-6

**Published:** 2025-10-24

**Authors:** Ruşen Ali Sayat, Özlem Bozo

**Affiliations:** Department of Psychology, Middle East Technical University, Ankara, Türkiye

**Keywords:** Meat-eating justifications, Speciesism, Meat-related cognitive dissonance, Social dominance orientation

## Abstract

**Background:**

People often avoid and try to stay ignorant of the negative aspects of meat production and consumption. When asked, people justify their meat consumption by emphasizing the necessity of meat for health, its desirable taste, naturalness, and normality. Relevant measures were developed to measure how much individuals rely on meat-eating justifications. Thus, the current work aims to translate and adapt the 4Ns scale by Piazza et al. (Appetite 91:114–128, 2015) and the Meat-eating Justifications Scale (MEJ) by Rothgerber (Psychol Men Masc 14(4):363–375, 2013) to Turkish to enable theoretical and applied studies of meat-eating in Turkish.

**Methods:**

The relevant measures are translated and adapted to Turkish by expanding the item pool. The data of the translated measures and other relevant variables is collected between May 22 and December 31, 2022 (*N* = 520). The sample is divided into two parts: the first subsample is used to conduct Exploratory Factor Analysis (EFA), and the other is used to conduct Confirmatory Factor Analysis (CFA). Finally, a correlation matrix is created with the finalized versions of the meat-eating justification measures and other variables of interest.

**Results:**

EFA for the 4Ns revealed a four-factor solution aligning with the original theoretical framework. Since the original measure accepted a single-factor solution, the current work improved on the original by reworking the low-loading items and introducing culture-specific items. After decisions about certain items, CFA is conducted, which indicated good fit (*χ*^*2*^(98) = 193.4, *p* < .05, *CFI* = .96, *RMSEA* = .061, [*CI* = .048, .074]), internal consistency reliability (Cronbach’s *α* = .95, McDonald’s ω_total = .95) and test–retest reliability (.92). EFA for MEJ indicated an eight-factor solution, where a subscale was discarded. CFA showed good fit (*χ2*(224) = 435.9, *p* < .05, *CFI* = .945, *RMSEA* = .06, [CI = .052, .069]), internal consistency reliability (Cronbach’s *α* = .89, McDonald’s *ω*_total = .91) and test–retest reliability (.89).

**Conclusion:**

Cross-cultural adaptation and validation of the meat-eating justification scales enable a better theoretical understanding of the psychology of meat-eating. This is demonstrated by replicating the strong associations between meat-eating, its justifications, social dominance orientation, speciesism, and masculinity in a different cultural context. This understanding is essential for prevention and intervention efforts to reduce meat consumption.

## Introduction

Consumption of meat has been brought into question by more individuals in recent years, whether due to ethical issues or environmental or health-related concerns [25]. Theoretically, in terms of cognitive dissonance, when individuals have conflicting attitudes and behaviors, they experience mental discomfort, which they are motivated to resolve in several ways [16]. Liking animals but eating their meat, or the "meat paradox," is an example [33]. Thus, it is expected for individuals who consume meat and have positive attitudes toward animals to experience such discomfort and become motivated to resolve it. Possible solutions are either changing the behavior or the attitude to align them or rationalizing meat-eating by adding new information or behavior to the equation.

Even though vegan/vegetarian diets have become more popular, these diets are still in the minority, which indicates that only a tiny proportion of meat-eaters that experience meat paradox resolve the cognitive dissonance of the meat paradox by adjusting their behaviors. It raises the question of how others handle this behavior-attitude inconsistency. One possibility is fluctuations in attitudes toward animals. On average, meat-eaters have a less positive attitude toward farm animals than those who do not eat meat [31]. Nonetheless, their attitude scores are still positive. This finding might beg whether eating meat leads to dissonance and whether meat-eating is rationalized through some strategies.

Bastian et al. [4] provided insight into these questions. Firstly, they cited the finding of Hoogland et al. [21] that meat-eaters dissociate the meat they eat from its source animal. Thus, eating meat might not lead to dissonance since eating meat does not make the source salient. Secondly, across three studies, they found that animals' mental capacity was negatively associated with their edibility. In addition, the moral relevance of the animal was increased as more capacity was attributed. Moreover, when the source was made salient, meat-eaters attributed less mental capacity to the animal, and attributing less mental capacity was related to less negative mood, which could be conceptualized as a strategy to resolve the discomfort of facing the meat paradox. Therefore, it is appropriate to ask what other strategies and rationalizations might be endorsed and which are the most common.

Examining the relation between meat consumption and other variables could be fruitful in finding such answers. For example, speciesism, which holds that species are not equal, is positively correlated with meat consumption, [12]. Similarly, it was found that meat consumption and social dominance orientation (SDO) were also positively correlated [6, 53]. SDO was coined by Pratto [43] and is an orientation characterized by a preference for hierarchy in society. Additionally, it was found that males have a higher meat consumption than females, and endorsement of masculinity was also associated with eating meat [46, 52]. Moreover, recent work shows children may justify eating animals differently than adolescents and adults [36]. Additionally, people may depend on mind denial more based on their openness/intellect or emotion regulation [49]. Thus, it could be argued that these variables and many others might have ideological parallels to the rationalizations of meat consumption. For example, a possible item from a speciesism scale, "humans are superior to other animals and have the right to use them as resources," could also be a rationalization for eating meat.

In this sense, Piazza et al. [42] asked the participants why they think it is okay to eat meat. Their theoretical framework was adapted from Joy [27], who suggested "3Ns of meat-eating justifications,"; beliefs that eating meat is natural, necessary, and normal. Justifications classified as "natural" include thoughts such as eating meat is an evolutionary part of human biology or it is God's way of creating humans. The second justification indicates that meat is "necessary" for physiological health and strength. Finally, justifications based on the notion that meat consumption is a norm in society are classified in the "normal" category. In addition to these categories, Piazza et al. [42] have suggested that there could be a fourth N: "Nice", which refers to the pleasantness of eating meat regarding its taste and other sensory factors.

They found that these four categories accounted for 83–91% of the responses in different samples. Then, they have developed a scale called "The 4Ns Scale" to measure how individuals endorse these rationalization categories. The scale was able to predict participants' scores on the Motivations to Continue to Eat Meat Scale, which was also developed within the scope of the same article. Moreover, the 4Ns endorsement was related to SDO, mind attribution, speciesism, and gender in the same direction as they are related to meat consumption. Another scale with similar premises was used to analyze the validity of the 4Ns Scale, which is the Meat-Eating Justification Scale (MEJ) by Rothgerber [46].

MEJ was developed to assess the types of justifications individuals use for eating meat to study differences in terms of gender and masculinity in an undergraduate student sample. It includes nine subscales (three items each) categorized as direct or indirect strategies. The latter includes avoiding thinking about how meat is obtained or avoiding thinking about the meat's source. In contrast, direct strategies include beliefs in the necessity of meat for strength and health or in the hierarchy between humans and animals. The study revealed that justifications endorsed by female students were more indirect while male students used more direct strategies. Moreover, it was found that direct strategies were positively correlated with masculinity, while indirect strategies were negatively correlated. Furthermore, the relation between gender and direct MEJ strategies became mostly unrelated when controlling for masculinity, which, as discussed earlier, is a critical ideology regarding meat consumption.

Piazza et al. [42] found that 4Ns and all direct strategies were significantly correlated since the 4Ns Scale was developed from the qualitative data that only included direct rationalizations due to the nature of the question they asked to the participants, which was why participants think it is okay to eat meat. The authors also aimed to assess how many of these direct strategies subscales might be subsumed by 4Ns categories. All nine categories were claimed to be subsumed by the subscales. For example, the religious justifications subcategory was suggested to be a part of the natural category of the 4Ns. However, none of the four items of the natural subscale refers to religion. It was suggested that since the operational definition of religious justification in MEJ refers to the naturality due to God's will that people have dominion over animals, the natural subscale was claimed to capture agreement with those justifications. It was logical in their case since these categories were highly correlated. Furthermore, their sample was comprised of individuals from diverse backgrounds where none of the religions had followers more than a quarter of the participants. However, the lack of religious justification items might prevent studies in different cultures and countries from accounting for the role of religion in meat-eating, which relates to the aim of this study.

This study aims to translate and adapt some of the mentioned measures to Turkish and its cultural context. Turkish context for the meat paradox could not be described because it has not been studied in Türkiye before, as the literature review revealed. It might be due to the lack of available data on vegan and vegetarian populations in Türkiye [1]. In terms of the context, religion might be one of the primary candidates for meat-eating justifications in Türkiye since 89% to 99.2% of the Turkish population follows Islam, according to different sources [39, 41]. In Islam, animal sacrifice is one of the main religious rituals with a dedicated religious holiday. This holiday is one of the two major religious holidays with a governmental recognition in Türkiye. Thus, this study also investigates whether the natural subscale would be able to capture religious justifications in a sample where meat-related worship is practiced. In addition, as a control variable, household income is included in the study to explore whether economic development of Türkiye influences meat consumption and meat-eating justifications across different income levels.

Several studies indicated a strong connection between perceptions of eating meat and masculinity in Turkish men. For example, Schösler et al. [47] found that in the Netherlands, among different ethnic groups, Turkish men had the strongest link in terms of the perceptions of meat and masculinity. In addition, a qualitative study conducted by Carpar [5] with male athletes found that eating meat was seen as a symbol of masculinity, and vegetarian/vegan diets were perceived to be feminine. Moreover, participants claimed that males who do not eat meat would be excluded from homosocial circles and activities such as having a barbecue. Thus, it is expected that 4Ns or the direct strategies of MEJ will be endorsed more by male participants. Furthermore, this relation is expected to be weaker when controlling for masculinity in the light of Rothgerber’s [46] study.

Thus, to investigate meat-eating, cognitive dissonance, and related justifications in Türkiye among meat-eaters, this study aims to translate and adapt the relevant measures, i.e., the 4Ns Scale and Motivations to Continue to Eat Meat Scale by Piazza et al. [42], and the Meat-Eating Justification Scale (MEJ) by Rothgerber [46], and the Mind Attribution Scale by Gray et al. [17] to Turkish. Finally, it is aimed to analyze whether findings from other countries and samples regarding SDO, speciesism, gender, and masculinity concerning meat-eating rationalizations in meat-eaters compared to vegetarians/vegans could be replicated in Türkiye.

## Method

### Recruitment

Five hundred twenty participants are recruited through convenience sampling through two different channels. The first channel was the study credit system of the institution and the rest of the participants are recruited through social media posts that included invitation to the study. Demographic details of the sample are presented in the results section.

### Measures

#### Demographic information form

In this form, participants were asked to report their age, gender, education, and household income. In addition, participants were asked if they had any physiological condition worth noting. Also, they were asked to report their diet type, i.e., if their diet is omnivore, partial vegetarianism, vegetarianism, or veganism.

#### The diet form

It was developed by the first author of the current study based on Rothgerber [46]. Participants filled out this form by indicating how many days a week they consume certain products listed in the form, including several types of meats, vegetables, and fruits.

#### The 4 N scale

It was developed by Piazza et al. [42] to determine how strongly individuals endorse common meat-eating justifications. Higher scores on the scale indicate stronger endorsement of meat-eating justifications. It is a 16-item scale measured on a 7-point (1 = strongly disagree, 7 = strongly agree) Likert scale. It is composed of four subscales, namely *necessary* (*α* = 0.91, e.g., "A healthy diet requires at least some meat"), *nice* (*α* = 0.87, e.g., "Meals without meat would just be bland and boring"), *natural* (*α* = 0.81, e.g., "Human beings naturally crave meat"), and finally *normal* (*α* = 0.54, e.g., "It is normal to eat meat"). Regarding the last subscale and its relatively low alpha coefficient, the authors suggested that further studies could test new items that paraphrase two of the items, "Not eating meat is socially unacceptable" and "Most people I know eat meat." These items loaded on their subscale with 0.33 and 0.40, while the other two items in the subscale had 0.71 and 0.77 loadings. The authors stated that these two problematic items were understood as inquiring about facts or observations rather than the participants' attitudes. In this light, the current authors included such replacements in the Turkish version as "It is normal that meat-eating is socially acceptable" and "Most people I know, no wonder why, eat meat." In the original study, inter-item correlations ranged between 0.62 and 0.75, and Cronbach's *α* of the full scale was 0.94. Test–retest reliability correlation was 0.93. There was a high correlation between 4 N scale scores and participants’ commitment to meat-eating (*r* = 0.85, *p* < 0.001), indicating predictive validity of the scale.

#### Meat-Eating Justification (MEJ) scale

The original scale was developed by Rothgerber [46] for another study that investigated the link between perceptions of meat-eating and perceptions of masculinity. MEJ Scale is a 9-point (1 = strongly disagree, 9 = strongly agree), 27-item Likert scale composed of nine subscales with three items each. These subscales are *pro-meat attitudes *(*α* = 0.77, e.g., "Meat tastes too good to worry about what all the critics say"), *hierarchical justification* (*α* = 0.71, e.g., "It's acceptable to eat certain animals because they're bred for that purpose"), *religious justification* (*α* = 0.83, e.g., "God intended for us to eat animals"), *health justification* (*α* = 0.87, e.g., "We need meat for a healthy diet"), *human destiny/fate justification* (*α* = 0.55, e.g., "Our early ancestors ate meat, and we are supposed to also"), *dichotomization* (*α* = 0.55, e.g., "It seems wrong that people in some cultures eat dogs and cats"), *dissociation* (*α* = 0.81, e.g., "I do not like to think about where the meat I eat comes from"), *avoidance* (*α* = 0.78, e.g., "I would have problems touring a slaughterhouse"), and *denial* (*α* = 0.71, e.g., "Animals don't really suffer when being raised and killed for meat").

These nine subscales are also grouped under two categories. Dissociation, avoidance, and denial are considered *indirect strategies*, while the remaining subscales -except dichotomization- are considered *direct strategies*. The Cronbach's *α* of the full scale was 0.85. Except for dichotomization, all other subscales were correlated with each other; direct and indirect strategies were negatively correlated and there were positive associations within themselves. In other words, a composite score could be achieved by reverse-coding the indirect strategies, where higher scores indicate higher endorsement of meat-eating justifications. In terms of validity, the direct strategies scales could predict beef consumption (all *r* scores were between 0.36 and 0.63 and all *p* values were smaller than 0.05).

#### Dimensions of mind attribution & mind attribution scale

Eighteen mind attribution dimensions were identified by Gray et al. [17] to describe the qualities based on which humans perceive the mind. According to the factor analyses, these dimensions were grouped under two categories, *experience* (11 items, e.g., hunger, joy) and *agency* (7 items, e.g., self-control, thought). In terms of the mind attribution scale, which is adapted from Piazza et al. [42], the dimensions determined by Gray et al. [17] are given to the participants as a list and they are instructed to imagine a cow. Then, they are asked to indicate how much they agree that a cow possesses those mental capacities on a 7-point Likert scale (1 = completely disagree, 7 = completely agree). The Cronbach's alpha of the scale was reported as 0.89.

#### Meat Commitment Scale (MCS)

MCS was developed by Piazza et al. [42], which is a 7-point (1 = strongly disagree, 7 = strongly agree) 7-item Likert scale that measures how committed individuals are to continue to consume meat. Higher scores on the scale indicate the participants are more committed to eating meat. An example item is "I would never give up eating meat". The internal reliability was 0.96, and the test–retest reliability was 0.93. This scale was developed, in part, to test the predictive validity of the 4 N scale. Thus, 4 N scale was argued to show the convergent validity of the MCS, since it was significantly associated with meat-eating justification endorsement of the participants (*r* = 0.85, *p* < 0.001).

#### Ambivalent speciesism scale

The scale was developed in Turkish by Altınal and Tekdemir [3]. It measures speciesism in three dimensions, which are *belief in human superiority*, *protective speciesism*, and *speciesism in language*. After reverse coding, higher composite scores indicate higher speciesism. It is a 7-point (1 = definitely disagree, 7 = definitely agree), 15-item Likert scale. The internal consistency reliability coefficient of the scale is 0.90. The protective speciesism subscale comprises eight items (*α* = 0.90, e.g., "We should support family farms and small-scale businesses, where they treat the animals better, instead of the meat industry"), the belief in the human superiority subscale comprises five items (*α* = 0.73, e.g., "Animals exist to serve human needs"), and speciesism in language subscale consists of two items (*α* = 0.75, e.g., "It is acceptable to call someone with animal names to insult them."). The scale showed convergent validity with another established speciesism scale (*r* = 0.57, *p* < 0.01, [6]).

#### New Social Dominance Orientation (SDO) scale

SDO Scale was developed by Ho et al. [20], which includes the researchers who coined the original term in Pratto et al. [43]. It is a 7-point (1 = definitely disagree, 7 = definitely agree), 16-item Likert scale with two subscales, which are *dominance* (SDO-D; *α* = 0.88, e.g., "Some groups of people are simply inferior to other groups") and *egalitarian* (SDO-E; *α* = 0.90, e.g., "We shouldn't try to guarantee that every group has the same quality of life"). While the first one refers to explicit support for the dominance of stronger groups over the others, the latter maintains a more implicit attitude that supports the same view. The overall reliability of the scale was reported as 0.93. Confirmatory factor analyses supported both a two-factor and a single-factor solution, which enables a composite score that is indicative of how strongly participants endorse the dominance orientation. After reverse coding, higher scores indicate a stronger endorsement of social dominance ideology. The scale was adapted to Turkish by Kaynak et al. [28]. While the overall Turkish scale has an internal consistency reliability coefficient of 0.87, SDO-E and SDO-D subscales' alpha coefficients are 0.83 and.72, respectively. Test–retest reliability of Turkish SDO scale is 0.92.

#### Religious orientation scale

The scale was developed by Allport and Ross [2]. It is a 5-point (1 = definitely disagree, 5 = definitely agree), 20-item scale with two subscales, which are *extrinsic* and *intrinsic* motivations of religiosity. By extrinsic, Allport and Ross [2] mean perception of religion as a tool for personal goals (*α* = between 0.76 and 0.85, 11 items, e.g., ""I go to church mostly to spend time with my friends."), whereas intrinsic motivation suggests that religion is the main goal (*α* = between 0.67 and 0.93, 9 items, e.g., "I enjoy reading about my religion"). The alpha values are reported in Donahue [14]. The scale was adapted to Turkish by Cirhinoğlu [7]. Since the original scale was developed for churchgoers, thus for Christians, the adaptation of the scale into the Turkish context required it to relate to Islam instead of Christianity due to the demographical and cultural differences. In Cirhinoğlu [7], there are 11 items for each subscale, where the internal consistency reliability coefficient of the intrinsic subscale was 0.87 and the extrinsic subscale was 0.60, and for the overall scale it was 0.90. Split half reliability was reported as 0.89.

#### Male role norms scale

The Male Role Norms Scale (MRNS) was developed by Thompson and Pleck [50] and adapted to Turkish by Lease et al. [32]. It is a 5-point (1 = disagree, 5 = agree), 26-item scale that is composed of three subscales, which are *toughness* (*α* = 0.82, e.g., "A man should never back down in the face of trouble"), *status* (*α* = 0.90, e.g., "The best way for a young man to get the respect of other people is to get a job‚ take it seriously‚ and do it well"), and *antifemininity* (*α* = 0.81, e.g., "If I heard about a man who was a hairdresser or a gourmet cook‚ I might wonder how masculine he was"). According to Lease et al. [32], the internal consistency reliability of the original scale ranges between 0.73 and 0.81 in different studies. Similarly, the Cronbach's alpha of the Turkish scale was reported as 0.82. However, confirmatory factor analysis results indicated that a four-factor solution could be a better fit for the Turkish version. Nevertheless, the current study used the composite score, where higher scores on the scale indicate stronger endorsement of traditional male role norms or masculinity [46]. In the Turkish version, the internal consistency reliability was 0.73 for the antifemininity subscale, 0.72 for the toughness subscale, and 0.82 for the status norm subscale. The overall internal consistency reliability score was reported in a separate study as 0.88. [19].

### Procedure

#### Translation

Permission to translate and use the Dimensions of Mind Attribution by Gray et al. [17] was obtained online via RightsLink by the Copyright Clearance Center. The rest of the measures, namely the 4Ns Scale and the Meat Commitment Scale by Piazza et al. [42], the Meat-Eating Justification Scale (MEJ) by Rothgerber [46] are made public domain by the authors via Measures Library by PHAIR (The Society for the Psychology of Human-Animal Intergroup Relations), and were translated into Turkish. All measures were translated by the present authors and two other researchers who were blind to the content of the study. Then, the authors and another independent researcher decided on the final versions of the items. In addition, all the measures were back-translated into English by another native Turkish-speaking researcher with English proficiency credentials, who was also blind to the content of the study. The authors then compared the original and the back-translated versions of the measures to detect any significant loss or change in the meaning of the items. In addition, during the initial translations, considering the original article's suggestions regarding the *normal* subscale of the measure, the authors wrote new items for the 4N Scale correspondingly. This process is detailed in the relevant section for the measure.

#### Data collection

Before the data collection, an institutional review board approval was obtained from the Human Subjects Ethics Committee of the Middle East Technical University (231-ODTUİAEK-2022). Data collection took place between May 22 and December 31, 2022. The participants were invited to the study via social media and the Sona System of the university. The purpose of the study was clearly stated prior to the study. Participants responded to the survey via Qualtrics. In the first session, they filled out all the measures included in the study in a counterbalanced manner. In the second session, they filled out the measures translated and adapted into Turkish by the present researchers. This additional session took place two to four weeks following the first session to determine the test–retest reliability of the measures. As incentives, a place for a raffle was granted to participants who participated in both sessions. The raffle reward was a 100 TL (6.5 USD) coupon that can be used in an online shopping website. Three coupons were granted to three participants after the raffle. For participants who participated via the research system of the university, course credit was granted, too.

#### Data analysis

All hypotheses of the study and the approach to analyze the hypotheses were decided prior to the data collection, and no modifications were made at any later stage of the study. Descriptive statistics for demographic information were calculated. Participants were randomly divided into two independent subsamples using SPSS software. Specifically, we selected a random 50% of cases via the “Select Cases” option using “Random sample of cases”, tagged them with a grouping variable, and assigned the remaining cases to the second group. We then analyzed the two groups separately using “Split File” function. To verify equivalence, we compared the groups on age, gender, education level, and the primary study variables; no significant differences emerged (all ps > 0.05). Exploratory factor analysis was conducted to determine whether paraphrased items in the 4Ns Scale have acceptable loadings to the "normal" subscale of the measure and whether the items created to consider the Turkish context fit their respective subscales and the overall scale. Confirmatory factor analyses were conducted for the adapted measures to determine whether the adapted versions have good fit. Internal consistency reliabilities were calculated for each measure and their subscales using Cronbach's alpha and McDonald’s omega. Test–retest reliability scores were determined by correlating the scores from the first and second sessions. In addition, construct validity was examined by analyzing the already established associations in the literature using adapted versions of the scales. The 4Ns Scale and MEJ's correlation with social dominance orientation, speciesism, and masculinity were examined for convergent validity. In addition, the relation between the 4N Scale and MEJ was analyzed since they are both measures of endorsement of meat-eating justifications. After controlling for income, the 4N Scale and MEJ scores were analyzed for concurrent validity regarding meat consumption and commitment to meat eating.

## Results

### Participants

The study included 520 participants. Of the whole sample, 189 participants (36.35%) accepted to participate in an additional session that took place after two to four weeks of first session, which was conducted to determine test–retest reliability of the translated measures in the study. The only inclusion criterion to participate in the study was to be 18 years old or older. The mean age of the sample was 25.43 (*SD* = 8.74; range = 18–71). In the sample, 331 participants were women (63.7%) and 185 participants were men (35.6%). While 3 participants reported their gender as other (0.6%), remaining one participant did not want to report gender (0.2%). In terms of educational characteristics of the sample, 356 participants were undergraduate students (68.6%), and 134 participants had undergraduate or graduate degrees (25.8%). Details can be found in Table 1. In terms of demographic variables, there was not any significant differences between test and retest samples.

**Table 1:** Demographic variables (*N* = 520)

Variable		f	Percentage
Gender	Women	331	63.7
	Men	185	35.6
	Other	3	.6
	Did not specify	1	.2
	Primary school degree	1	.2
	Secondary school degree	1	.2
	High school degree	27	5.2
Education	Undergrad student	357	68.7
	Undergrad degree	68	13.1
	Grad student	40	7.7
	Grad degree	26	5.0

*f means frequency*

### The 4Ns of meat-eating justifications

To be able to conduct both exploratory factor analysis (EFA) and confirmatory factor analysis (CFA) for the adapted measures, the sample was randomly divided into two equal-sized samples. The random samples did not differ from each other significantly on any of the demographical variables. Exploratory factor analysis was conducted using jamovi software with the Factor module. Oblimin rotation was utilized due to the relatedness of the theoretical constructs within the scale [38], p. 325). Maximum likelihood rotation was used because, theoretically, the number of factors was already known, which eliminates the need for the ability to recover weaker factors [11]. Also, considering the number of items, the sample size was large enough to take advantage of more stable loading estimates of the maximum likelihood method for the following confirmatory factor analysis [8]. However, it should also be noted that multivariate normality was not ultimately achieved in the sample (as it will be apparent in the CFA with Mardia’s *Z* calculations), which is suggested to be a reason to use principal axes factoring over maximum likelihood extraction [8].

EFA indicated that a four-factor solution was possible based on parallel analysis. Based on eigenvalues greater than 1, the analysis recommended a two-factor solution. Since, theoretically, the scale was built on four factors that reflect a single construct, either a single factor or four factor solution made the most sense. Thus, the four-factor solution was accepted by the authors. The initial loadings of the items to four factors can be found in Table 2.

**Table 2:** Initial factor loadings for the 4Ns in exploratory factor analysis

	Factor					
	1	2	3			4
Item 1		0.731				
Item 2	0.489					
Item 3						0.517
Item 4						0.571
Item 5	0.316					0.568
Item 6	0.909					
Item 7	0.888					
Item 8	0.867					
Item 9	0.774					
Item 10	0.378					
Item 11		0.771				
Item 12			0.361			
Item 13		0.616				
Item 14		0.915				
Item 15	0.370		0.399			
Item 16		0.637				
Item 17			0.923			
Item 18			0.608			
Item 19			0.855			
Item 20			0.728			

Maximum likelihood extraction method was used in combination with oblimin rotation

The foremost exploration was on the items written from scratch. To strengthen the adaptation of the scale to the Turkish context, one item for each of the four subscales was written. Out of these four items, the item written for the normal subscale (item 15) did not load on the respective subscale. Moreover, it cross-loaded on two other scales. Thus, this item was removed from the scale. The item written for the necessary subscale (item 10) loaded on the correct subscale with a small albeit acceptable loading (0.38) but also cross-loaded on two other subscales. Therefore, it was also removed from the scale. Additionally, from the original scale, the direct translation of the item (item 12), "It is abnormal for humans not to eat meat" did not load on the correct subscale and loaded on the "nice" subscale (0.36). Since there is no logical reason to expect that item to relate to "nice" subscale, this item was also removed from the scale.

There were other unexpected results regarding item loadings in the exploratory factor analysis. First, the direct translation of the item "Meat is delicious" did not load on the "nice" subscale. This outcome is baffling because the item could be considered the most representative item of the subscale. Moreover, this item loaded on the "normal" subscale with a loading of 0.64. Theoretically, there is no ground to expect this item to load on the "normal" subscale since taste should represent a subjective opinion rather than the perception of what is or should be normal. Thus, this item is removed from the scale as well.

Additionally, the direct translation of the item "It is only natural to eat meat" loaded on the "normal" subscale with loading of 0.72. This item was added to the normal subscale as it had a high loading (0.72) and doing so made theoretical and logical sense since in Turkish meaning of natural and normal are often used interchangeably. Finally, the item "It is unnatural to eat an all plant-based diet" loaded on the necessary subscale rather than the natural subscale with loadings of 0.49 and 0.25, respectively. Considering that the difference between loadings was larger than 0.20, the item did not cross-load [15]. Thus, the item is included in the necessary subscale. After the changes, the overall scale comprised four subscales and 16 items (Appendix A). The subscales and their item counts are as follows: "normal" with four items, "necessary" with five items, "natural" with three items, and "nice" with four items.

In addition, the two lowest-loading items in the original scale ("Not eating meat is socially unacceptable" and "Most people I know eat meat"), which belonged to the normal subscale, were changed in the current study to increase their loadings. The first item (item 11) was changed as " It is normal that meat-eating is socially acceptable." The analysis indicated that this item loaded on the normal subscale with 0.77. The other item was changed as " Most people I know no wonder eat meat." This item (item 13) also loaded on the normal subscale with 0.62.

Next, a confirmatory factor analysis (CFA) was conducted with the other half of the initial sample using the EQS software to confirm that the four-factor solution presents a good fit. The results obtained from the robust method are reported since Mardia's *Z* (41.3) was larger than 5 [29]. The results indicated that the model had a good fit (*χ*^*2*^(98) = 193.4, *p* < 0.05, *CFI* = 0.96*, SRMR* = 0.046, *RMSEA* = 0.061, [*CI* = 0.048, 0.074]).

It is desired for the chi-square test to be insignificant to support that model and data do not significantly differ. However, due to the nature of significance testing, the results are likely to be significant when larger sample sizes are present [24]. In this situation, it is recommended that the estimate found by dividing the chi-square value by degrees of freedom to be lower than two to support an acceptable fit of the model [30], which is the case in the current analysis (193.4/98 = 1.97). In terms of CFI, it is suggested that CFI values of 0.95 and higher indicate a good fit [48]. For RMSEA, values below 0.05 indicate good fit, values between 0.05 and 0.08 suggest acceptable fit, values between 0.08 and 0.1 are thought to reflect marginal fit, and values above 0.1 reflect poor fit [55]. As the results indicated, these recommendations were met.

Test–retest reliability of the final version of the scale was 0.92, and its Cronbach's alpha was 0.95 and McDonald’s ω_total was 0.95, which indicates good internal reliability [40]. Subscales also showed good internal consistency. The normal subscale had a Cronbach's alpha of 0.87 (McDonald’s ω_total was 0.87), and it was 0.93 (McDonald’s ω_total was 0.94) for the necessary subscale, 0.84 (McDonald’s ω_total was 0.85) for the natural, and 0.88 (McDonald’s ω_total was 0.89) for the nice subscale. In terms of construct validity, the current scale had high correlation (*r* = 0.81, *p* < 0.05) with the adapted version of the Meat-Eating Justifications Scale (MEJ), whose psychometrics are discussed in the following section. Thus, high correlation between the two scales that measure the endorsement of meat-eating justifications supported that the scales measure the same construct. In terms of convergent validity of similar constructs, findings regarding correlates of meat-eating justifications, such as social dominance orientation and speciesism, were replicated in the Turkish context with the adapted scale.In terms of social dominance orientation, it was found that higher endorsement of meat-eating justifications was positively associated with higher social dominance orientation scores (*r* = 0.41, *p* < 0.05). In addition, 4Ns scores correlated significantly and positively with the endorsement of masculine ideology (*r* = 0.56, *p* < 0.05) and speciesism (*r* = 0.77, *p* < 0.05). Also, meat-eating justifications correlated significantly and negatively with the attribution of mind to cows (*r* = −0.32, *p* < 0.05). In addition, this study found evidence that meat-eating justifications and religiosity (measured for Islam) are associated as well (*r* = 0.24, *p* < 0.05). For concurrent validity, it was found that commitment to continue eating meat (*r* = 0.78, *p* < 0.05) and meat consumption (*r* = 0.45, *p* < 0.05) have positive and significant correlation with meat-eating justification scores, which are criterion variables that were expected to be associated with endorsement of meat-eating justifications when measured at a time. Adding income (dummy coded) as a control to the regression where endorsement of the 4Ns predicts meat consumption did not result in a meaningful difference in model comparison (*F*(3,515) = 0.79, *p* = 0.5).

### Meat Eating Justifications Scale (MEJ)

Exploratory factor analysis (EFA) was conducted using JAMOVI software with factor analysis package. Oblimin rotation and maximum likelihood rotation were used for the same reasons as the previous analysis for the 4Ns scale. EFA indicated a three-factor solution based on parallel analysis and eigenvalues larger than 1. Since theoretically, the scale was built on either two main factors of direct and indirect strategies or the nine factors, the three-factor solution suggested by the analysis was evaluated considering the two-factor theoretical structure of the original scale as the relevant theoretical structure. Moreover, extracting nine theoretically related factors from 27 items in exploratory factor analysis might not be realistic because too few items are available for each factor, which are already numerous. Thus, exploratory factor analysis was conducted to determine whether the two-factor solution could be supported, whether items load on the factors without cross-loading, and whether the third factor can make sense theoretically. Initial loadings can be found in Table 3.

**Table 3:** Initial factor loadings of Meat-Eating Justifications Scale (MEJ)

	Factor		
	**1**	**2**	3
Item 1	0.711		
Item 2		0.466	
Item 3	0.468	0.404	
Item 4	0.475		
Item 5			0.631
Item 6		0.849	
Item 7			0.801
Item 8	0.905		
Item 9	0.795		
Item 10	0.602		
Item 11		0.578	
Item 12	0.602		
Item 13			
Item 14			0.686
Item 15		0.892	
Item 16			0.525
Item 17	0.594		
Item 18	0.622		
Item 19	0.632		
Item 20		0.399	
Item 21		0.803	
Item 22			
Item 23			0.840
Item 24		0.662	
Item 25			0.762
Item 26	0.957		
Item 27	0.809		

Maximum likelihood extraction method was used in combination with oblimin rotation

The first issue addressed was items that did not load on any factor. Two items from the dichotomy scale did not load on any of the factors, and one loaded on direct strategies. This problem was also present in the original scale, as the dichotomy subscale had low internal reliability and low correlation with the rest of the scale. Moreover, even if the analysis was forced to two factors, these items either did not load on any of the factors or cross-loaded weakly on both factors. Thus, these two items were removed from the scale. Since the third item of the dichotomy subscale could not be included in other subscales theoretically, and to enable the researchers use the scale with eight remaining subscales, it was also removed from the scale.

Like Piazza et al. [42], who also used MEJ in their scale development study, the current paper found three factors for MEJ as well, two of which represent direct and indirect strategies and a third factor composed of religious justifications. Two of the three items of denying animal suffering and an item of hierarchical justifications were also factored with all of religious justifications items. However, since half of these six items also cross-loaded on the direct strategies subscale, a reasonable distinction of this factor could not be made. With the remaining eight subscales and 24 items (Appendix B), a confirmatory factor analysis with eight factors was conducted using the EQS software on the other half of the initial sample.

The results obtained from the robust method are reported since Mardia's *Z* (114.3) was larger than 5 [29]. The results indicated that the eight-factor model had a good fit to the second half of the sample (*χ2*(224) = 435.9, *p* < 0.05, *CFI* = 0.945, *SRMR* = 0.08, *RMSEA* = 0.06, [CI = 0.052, 0.069]). As discussed earlier, due to the sample size chi-square significance testing might be significant even if the model has good fit, which is indicated by an insignificant result. Dividing the chi-square value by degrees of freedom yielded a value of 1.95, which is under the recommended rule of thumb of 2 [30]. As discussed earlier, other fit indices are also within either good or acceptable thresholds.

In terms of internal consistency, Cronbach's alpha coefficient of the MEJ was 0.89 and the McDonald’s ω_total was 0.91. Internal consistency coefficients of the subscales were also calculated. Since Cronbach’s alpha and McDonald’s ω_total scores were virtually the same, they are not reported separately. They were 0.86 for pro meat, 0.75 for denial of suffering, 0.85 for hierarchy justification, 0.80 for dissociation, 0.86 for religious, 0.75 for avoidance, 0.85 for health justifications, and 0.80 for human destiny (0.40 for dichotomy). Test–retest reliability coefficient of MEJ was also good (*r* = 0.89).

For the convergent validity, as discussed earlier, the correlation between the current scale and the 4Ns scale that aim to measure the same construct was 0.81. In terms of other correlates discussed in the earlier section about convergent validity and concurrent validity of the 4Ns scale, results were similar in terms of significance, but correlations were visibly smaller (Table 4). In addition, the findings from the original study regarding gender and utilization of direct or indirect strategies were replicated. The results indicated that women compared to men utilized indirect strategies significantly more (*X*^*2*^(1,516) = 92.9, *p* <. 001), and men compared to women utilized direct strategies significantly more (*X*^*2*^(1,516) = 33.6, *p* <. 001).

**Table 4:** Correlation Matrix of the adapted measures and the other study variables

	The 4Ns	MEJ	SDO	Age	Rel. Or.	Meat Con.	Speciesism	Commit.	Mental C.	Masculinity
The 4Ns	(.95)									
MEJ	0.81^***^	(.89)								
SDO	0.41^***^	0.32^***^	(.93)							
Age	0.11^*^	0.13^**^	−0.09^*^							
Rel. Or	0.24^***^	0.40^***^	0.05	0.28^***^	(.87)					
Meat Con.	0.45^***^	0.35^***^	0.30^***^	−0.12^**^	−0.03					
Speciesism	0.77^***^	0.69^***^	0.42^***^	0.05	0.22^***^	0.40^***^	(.90)			
Commit.	0.78^***^	0.64^***^	0.40^***^	0.05	0.12^*^	0.50^***^	0.64^***^	(.95)		
Mental Cap.	−0.32^***^	−0.34^***^	−0.17^***^	−0.13^**^	−0.14^**^	−0.11^*^	−0.30^***^	−0.18^***^	(.92)	
Masculinity	0.56^***^	0.54^***^	0.37^***^	0.26^***^	0.30^***^	0.30^***^	0.47^***^	0.53^***^	−0.25^***^	(.82)

*Note 1*. *p <.05, **p <.01, ***p <.001

*Note 2*. MEJ (Meat-eating Justifications Scale), Rel. Or.(Religious Orientation), Meat Con.(Meat Consumption), Commit.(Commitment to meat consumption), Mental Cap.(Mental capacity attributed to farm animals)

*Note 3*. Scores in the parentheses along the diagonal indicate Cronbach’s alpha values for the corresponding measures

### Correlations between the adapted measures and the other variables in the study

As one of the primary purposes of the study, the correlates of meat-eating and endorsement of meat-eating justifications were analyzed to explore the nature of their relations in the context of Türkiye. Thus, a correlation matrix including the endorsement of the 4Ns, MEJ (meat-eating justifications scale), SDO (social dominance orientation), age, religious orientation, mind attribution to farm animals, meat consumption, commitment to meat, masculinity, and speciesism was created (Table 4).

For categorical variables, i.e., gender, education, and income, Kruskal–Wallis test was used. *T*-tests and ANOVAs could not be utilized because the data did not meet normal distribution assumptions for the variables to be analyzed (all Shapiro–Wilk p’s < 0.05). For gender, the results indicated that men endorse the 4Ns more (*X*^*2*^(1, 516) = 55.7, *p* < 0.001), have significantly higher scores on MEJ (*X*^*2*^(1, 516) = 4.62, *p* < 0.05), and have significantly higher social dominance orientation (*X*^*2*^(1, 516) = 32.3, *p* < 0.001), but women and men did not differ in terms of mind attributed to farm animals (*X*^*2*^(1, 516) = 0.008, *p* = 0.93). In addition, women reported significantly less meat consumption (*X*^*2*^(1, 516) = 43.6, *p* < 0.001) and less commitment to continue eating meat (*X*^*2*^(1, 516) = 86.5, *p* < 0.001). Also, women endorsed masculinity significantly less than men and showed significantly less speciesism (*X*^*2*^(1, 516) = 53.7, *p* < 0.001).

In this study, income was collected to account for the economic ability to purchase and consume meat. As a control, as reported earlier, income did not explain a significant portion of the variance in the association between meat-eating justifications and meat consumption (*F*(3, 515) = 0.79, *p* = 0.51). Moreover, the Kruskal–Wallis test for meat consumption and income was not significant (*X*^*2*^(3, 516) = 3.95, *p* = 0.27).

For education, participants who reported high school degree or lower degrees were grouped since only two participants reported lower degrees. Kruskal–Wallis test revealed that meat consumption differs significantly between the education levels (high school degrees or lower degrees, undergraduate students, undergraduate degrees, and graduate students/graduate degrees) of participants (*X*^*2*^(3, 516) = 9.1, *p* < 0.05). Dwass-Steel-Critchlow-Fligner (DSCF) pairwise comparisons showed that undergraduate students had the highest frequency of meat consumption; and compared to participants with bachelor’s degrees, they consumed more meat (*W* = −3.8, *p* < 0.05). The other pairwise comparisons were insignificant. Similarly, endorsement of the 4Ns differed significantly between different educational backgrounds (*X*^*2*^(3, 516) = 11.6, *p* < 0.05). DSCF pairwise analysis indicated that the only significant difference between education levels was between participants with bachelor’s degrees and participants who have graduate degrees or are currently studying in graduate programs, where the graduate education group endorsed the 4N’s significantly less (*W* = −3.68, *p* < 0.05). MEJ scores also differed across education levels (*X*^*2*^(3, 516) = 11.2, *p* < 0.05). For MEJ scores, pairwise comparisons showed that participants with bachelor’s degree scored significantly higher than undergraduate students on MEJ (*W* = 3.99, *p* < 0.05).

In terms of social dominance orientation, education levels differed significantly (*X*^*2*^(3, 516) = 13.7, *p* < 0.05). Pairwise analysis indicated that undergraduate students had the highest SDO, and they significantly differed from graduate level students (*W* = −4.8,* p* < 0.05). Other pairs were insignificant. For endorsement of masculinity, education was significant (*X*^*2*^(3, 516) = 32.6, *p* < 0.001), and both undergraduate students (*W* = 7.4,* p* < 0.001) and graduate students and participants with graduate degrees (*W* = −6.8,* p* < 0.001) endorsed masculinity significantly less than the participants with bachelor’s degree. Finally, speciesism did not differ concerning education (*X*^*2*^(3,516) = 3.8, *p* = 0.28).

Finally, for religious orientation, whose measurement was included in the study with the instructions that required participants to fill in the questionnaire if they felt items were applicable since the measure was written for individuals who follow Islam. Thus, out of 520 participants in the study, 409 participants filled out this questionnaire. Therefore, the analyses regarding religious orientation are conducted in two ways. Firstly, the participants who filled in the questionnaire are compared with individuals who did not fill out the scale. Then, the participants' scores who filled out the questionnaire are included in the correlation matrix mentioned earlier (Table 4). Participants who did and who did not fill out the questionnaire did not differ in terms of their meat consumption in the Kruskal–Wallis test (*X*^*2*^(1, 516) = 0.16, *p* = 0.69), commitment to meat consumption (*X*^*2*^(1, 516) = 0.31, *p* = 0.58), speciesism (*X*^*2*^(1, 516) = 1.4, *p* = 0.24), and endorsement of the 4Ns (*X*^*2*^(1, 516) = 3.2, *p* = 0.07). However, they differed in terms of the scores on MEJ (*X*^*2*^(1, 516) = 8.2, *p* < 0.05) and mind attribution to animals (*X*^*2*^(1,516) = 6.0, *p* < 0.05). In terms of the correlation matrix, religious orientation was not associated with meat consumption (*p* > 0.05). However, religious orientation was significantly and positively correlated with commitment to meat consumption (*r* = 0.12, *p* < 0.05), endorsement of the 4Ns (*r* = 0.24, *p* < 0.001), and MEJ (*r* = 0.40, *p* < 0.001).

## Discussion

Meat consumption has long been a concern for animal rights. With the expansion of animal agriculture in the last century, it has become more concerning due to greenhouse emissions, health risks, and related expenditures [45]*.* Such a range of concerns attracted many people to stand against animal agriculture [51]. However, it is also the case that animal products are becoming more popular, or at least stay popular around the globe [37, 54]. In this sense, the literature on justifications to continue meat consumption was explored in Türkiye, which had been virtually unexplored until the current study.

To enable such exploration, the researchers translated and adapted two assessment tools, which are the 4Ns of Meat Consumption by Piazza et al. [42] and the Meat-Eating Justifications Scale (MEJ) by Rothgerber [46]. Regarding the first one, the current study added four items, one for each of the four subscales, to the item pool that emphasize the Turkish context. Exploratory factor analysis yielded a four-factor solution, which aligns with the original scale's theoretical framework. However, certain items loaded on unexpected factors. Six decisions were made about these items based on theoretical and statistical information.

First, the item (item 15) written for the normal subscale (Guest tables must include a meat dish) for the current study was removed from the scale. The item was written initially to emphasize the normative role of meat in daily life without crossing the borders of the other subscales. In addition, by including a contextually relevant behavior and phrasing it in a norm-signaling tone about meat consumption, it was aimed to create an item that simultaneously could be agreed upon by participants who endorse the normal subscale of meat-eating justifications and avoid writing an item that is answered the same by the majority of the participants since the item states an observation of a norm. However, the item did not load to normal subscale but cross-loaded to two unintended subscales (necessary and nice). The reason could be that the norm is not as adhered to as thought by the researchers (*M* = 3.15, *SD* = 1.88, overall normal subscale *M* = 5.41, *SD* = 1.29, overall measure *M* = 4.22, *SD* = 1.36). Since the item cross-loaded on two unintended subscales, the issue regarding this item was resolved by omitting the item from the scale.

Second, the item (item 10) written for the necessary subscale (Meat dishes are a source of healing) was removed from the scale. Even though the item loaded on the correct subscale (0.36), it also loaded on the normal (0.23) and the natural (0.26) subscales. Even though the latter two loadings are below the rule of thumb value of 0.30 since they violate another rule of thumb about cross-loadings, there should be more than a 0.20 difference between cross-loadings [23],the item was removed from the scale.

Third, the translation of item 12 (It is abnormal for humans not to eat meat) from the original scale was removed because it loaded only on the nice subscale (0.36). Since there is no direct or inferable meaning about the desirability of meat, this item is removed from the scale. However, in terms of why the item did not load on the correct subscale, it could be argued that the Turkish wording of the item might not have been the easiest to understand at first read due to pseudo double negative wording that stems from the prefix "ab-." Especially when considering that prefixes are not native to Turkish, which might have exacerbated the possible confusion in meaning [18], p. 63).

Four, the item from the nice subscale (Meat is delicious) was removed from the scale because it loaded on the normal subscale (0.64), and any theoretical justifications could not be made to include the item in the normal subscale. The main reason was that since items in the normal subscale refer to societal and normative views on meat and the items of the nice subscale include more subjective opinions on the taste and desirability of meat, the subscales are innately aimed at measuring two relatively opposing points of views on meat consumption whether those views align or not.

Five, the direct translation of the item "It is only natural to eat meat" from the natural subscale was transferred to the normal subscale. Even though the translated item explicitly includes the keyword “natural” in the Turkish version, the item also loaded on the normal subscale. It might be argued that since in Turkish “natural” and “normal” are used interchangeably, even though they have separate meanings as well, the item could have been understood by the participants as a replicate of the item "it is normal to eat meat." Thus, this item was added to the normal subscale as it had a high loading (0.72) on the subscale, and doing so made both theoretical and logical sense.

Finally, the translation of the item from the natural subscale "It is unnatural to eat an all plant-based diet" was transferred to the necessary subscale. This decision was made because the item loaded on the natural subscale (0.49) and did not cross-load to other subscales. In terms of theoretical reasoning, the item included the Turkish word "beslenme" (means eating, nutrition, and diet), which was included to correspond to "eat" in the original item. However, the word "beslenme" might have had connotations with the concept of health-related aspects of diet; thus, the item might have been represented among meat-eating justifications concerned with the necessity of eating meat for health. Therefore, we included this item in the necessary subscale.

Concerning the original measure, the current study also translated and adapted certain items with intentional changes, which were changes in the two lower-loading items in the normal subscale as suggested by the original researchers [42]. First, the original item “Most people I know eat meat” was translated as “Most people I know no wonder eat meat”. With this change, it was aimed that participants with most of their social milieu composed of meat-eaters could be distinguished from each other, since in Türkiye, having a social circle that is not meat-eater majority would be exceptional. The results indicated that the item loaded on the correct subscale with an initial loading of 0.62. Thus, it could be argued that adding a more subjective component to the original item worked as intended.

Moreover, the original item “Not eating meat is socially unacceptable” was translated as “It is normal that meat-eating is socially acceptable”. In the original study, a possible reason for the item's low loading was its low endorsement. With this adaptation, the meaning of the item was shifted from “abnormality of not eating meat” to “how normal it is that society accepts meat-eating as an acceptable behavior”, which is a change that aims to increase the endorsement of the item while preserving its subscale. While it is known that meat-eaters can turn away from the idea of stopping meat consumption because their behaviors would be perceived as abnormal [34], it could be the case that there is a perceived distance between what is abnormal and unacceptable. In other words, to increase the endorsement of the item, the focus was shifted from not eating meat to eating meat. Thus, the item was paraphrased to account for participants' views on how meat-eating is acceptable rather than how out of the norm it is not to eat meat. The results indicated that the item loaded on the normal scale with an initial loading of 0.76. Thus, the item rework was statistically successful. However, there could still be an argument for the necessity of an item that emphasizes the nonnormality of not eating meat and its consequences for participants' social functioning as a justification for continuing to eat meat.

After the abovementioned chages, the overall scale had satisfactory reliability and validity values, as reported in the results section. In terms of contributions of the current study to the literature, adapting the scale to the Turkish context will enable future studies that examine different facets of meat consumption in Türkiye. Furthermore, cross-cultural studies that include meat-eating justifications can now have Turkish samples. Also, the addition of context-related items increased the reliability and validity of the adaptation since it was statistically and logically sound to include two out of four items in their respective subscales. Moreover, a similar argument could be made about paraphrasing the two normal subscale items in the original scale. In terms of removed items, future attempts to increase psychometric properties of adaptation might try to account for two subscales that did not eventually receive context-specific items, i.e., normal and the necessary subscales.

The Meat-eating Justifications Scale (MEJ) required fewer decisions than the 4Ns Scale. The only change between the adapted and the original version is that the dichotomy subscale is omitted from the scale since two of the three items of the subscale did not load on the scale, even if a single-factor solution was forced. The remaining item of the subscale was also removed since a subscale has to include at least three items, and the item would be left out on a possible eight-factor solution that could be tested with confirmatory factor analysis.

The three-factor solution revealed by the exploratory factor analysis, was not accepted. The first two subscales were known as direct and indirect strategies from the original scale. The third factor included two items from denying animal suffering and all items of hierarchical justifications and religious justifications. This factor was evaluated on the argument that common Islamic beliefs that animals are created to serve humans and that God prevents animals from suffering and feeling pain when killed for human benefit might have led these factors to be grouped [10, 44], p. 233). However, since most of these items also cross-loaded on the direct strategies factor as theorized by the original measure, this factor was not accepted as a third, separate factor. In this sense, the adapted measure was evaluated either on a two-factor or an eight-factor basis.

As reported in the results section, the current paper also explored various correlates of meat-eating justifications by utilizing the adapted measures. In terms of income, it was expected for income to account for some variance in the correlation between endorsement of meat-eating justifications and meat consumption. It was thought that although participants might endorse meat-eating justifications on a high level, they might not be able to afford meat as frequently as high-income participants. However, results indicated that income did not account for a significant variance in the abovementioned correlation. Moreover, meat consumption frequency did not differ depending on income. Thus, it might be argued that income does not have a significant role in how often people consume meat or how much they endorse meat-eating justifications. Further analysis, on the other hand, indicated that there was a significant difference between income levels in terms of how often participants consumed red meat (*X*^*2*^(3, 516) = 8.5, *p* < 0.05). It was found that highest income participants consumed red meat significantly more often than lowest income participants (*W* = 3.94, *p* < 0.05). Thus, even though frequency of overall meat consumption and endorsement of meat-eating justifications did not differ depending on income, the frequency of which type of meat is consumed might change. Moreover, it might be the case that higher-income participants consume a higher volume of red meat with a similar frequency of consumption to lower-income participants.

Considering the correlation matrix (Table 4), it could be argued that, similarly to previously studied contexts, in the Turkish sample, higher meat consumption and endorsement of meat-eating justifications were significantly associated with higher speciesism, higher endorsement of masculinity, more social dominance orientation, and less mental capacity attribution to farm animals. Thus, the current study provided the groundwork for contextual and cross-cultural applicability of previous work in the literature, such as the role of social dominance or speciesism in generalized prejudice [6, 13]. Moreover, the current work contributed to the literature by introducing religious orientation to the variables studied concerning meat consumption and meat-eating justifications. The results indicated that higher overall religious orientation was associated with significantly higher endorsement of meat-eating justifications, masculinity, higher speciesism, more commitment to meat, and less mind attribution to farm animals. However, it was not associated with meat consumption or social dominance orientation. Thus, even though the data did not reveal if individuals who are Muslim differ from others who are not in terms of endorsement of meat-eating justifications and other study variables, the results indicated that being more oriented towards Islam is associated with more commitment to meat consumption and endorsement of meat-eating justifications. Even though meat consumption was not related to religious orientation, due to other significant associations, it could be argued that individuals with higher religious orientation might be more challenging to persuade to give up meat consumption. Nonetheless, there could be interventions directed at individuals with higher religious orientations. For example, according to Dahlan [9] there are some muslim groups arguing that if animals suffer throughout their lives their meat would not be halal, which would be an argument to oppose the animal agriculture industry from an Islamic point of view.

Regarding results about gender, it could be argued that previous findings obtained from Western samples were replicated in Türkiye. Compared to men, women reported significantly less meat consumption, endorsed meat-eating justifications less, were less committed to meat consumption, had less social dominance orientation and speciesism. Regarding the meat-eating justifications scale, the results indicated that women use more indirect strategies and men use more direct strategies, which replicates Rothgerber [46]. Thus, future attempts to reduce meat consumption could be informed by this finding in the sense that specific justifications could be targeted and confronted depending on the participants' demographic characteristics.

In terms of education, it was found that undergraduate students consume meat more frequently than participants with bachelor’s degrees. It could be argued that due to dining halls, students might have more frequent access to meat than graduates. Thus, initiatives such as plant-based nudging in dining halls (e.g., making plant-based options the default choices and making plant-based options more visible) could be very efficient in reducing meat consumption for undergraduate students [35]. Moreover, it was found that graduate students or participants with graduate degrees endorsed the 4Ns significantly less than participants with bachelor's degrees. Additionally, it was found that undergraduate students endorsed meat-eating justifications significantly less than the participants with bachelor’s degrees. Thus, it might be the case that the active status of studentship at undergraduate or higher levels is associated with less endorsement of meat-eating justifications.

Age could also offer an explanation since active undergraduate students are mainly composed of younger participants compared to participants who hold a bachelor’s degree in the sense that age was negatively and significantly correlated with meat consumption and positively with the endorsement of 4Ns, which could explain both findings (Table 4). Thus, the role of education in meat-eating and meat-eating justifications was investigated by adding age as a control variable, which resulted in a nonsignificant difference in model comparisons (all *p*’s > 0.05).

In terms of other associations, a striking finding was that neither education nor age was associated with speciesism and commitment to meat consumption, which indicated that speciesism and commitment to meat do not follow the trend of having less endorsement with younger age or higher education. Thus, it could be argued that the current education system or younger generational values in Türkiye lack awareness about speciesism, which should be addressed since higher speciesism is associated with many undesirable outcomes, such as worse attitudes toward animals and their rights, and higher generalized prejudice [6, 26].

### Implications

The results have many methodological, theoretical and practical implications for animal product consumption literature. Firstly, even though the four facets of meat-eating justifications were distinguished in the analyses, the stronger evidence points out to the uniformity of the meat-eating justifications construct. This means that researchers that are more interested in the subscales of the 4Ns than the overall endorsement of the meat-eating justifications are recommended to apply bifactor models and consider adapting the already existing example of this, which is the Motivations to Eat Meat Inventory [22].

Theoretically, most of the exploratory literature regarding meat-eating and speciesism, which comes from the western countries, is replicated in Türkiye. Namely the gender differences in meat consumption, commitment to meat, endorsement of meat-eating justifications, social dominance orientation and speciesism along with the interrelationships among these variables. Thus, this study adds to the cross-cultural applicability of these constructs and theories. Differently, religious orientation was also included in the study, and it is associated with higher endorsement of meat-eating justifications and stronger commitment to meat consumption but not with actual meat consumption. Thus, individuals who have higher religious orientation appear to be a more resistant demographic for behavior change interventions, despite not consuming more meat overall. On the other hand, undergraduates may be a particularly promising group for interventions since they reported consuming meat most frequently while endorsing fewer justifications. This pattern suggests that interventions like implementing plant-based defaults or nudges in university dining settings could effectively decrease meat consumption.

Lastly, the absence of significant correlations between age or education with speciesism and commitment to meat may indicate that current generational shifts and education system are insufficient in addressing this behavioral and attitudinal domain Thus, conscious educational efforts that address speciesism and animal product consumption appear beneficial if not necessary.

### Limitations

Even though there is strong construct-level reliability and validity, confidence in the subscales in the 4Ns is constrained by the limitations of the study. Firstly, the sample was obtained through convenience sampling using social media platforms and the student credit system within the institution, which showed a bias for female participants. Thus, this sample may not be representative of general Turkish population or subgroups within. Secondly, the exploratory and confirmatory factor analyses are conducted with the split halves of the same sample. These limitations coupled with the fact that the four-subscale structure of the 4Ns found in this study deviates from the original measure require caution against the utilization of subscales in future studies without replications in different samples. Researchers specifically interested in subscale-level interpretation may consider using the Motivations to Eat Meat Inventory [22], however, this would also require translation and adaptation to the Turkish context. Despite these limitations surrounding sampling and the subscales, this study offers a strong introduction of prominent measures in animal product consumption literature to the Turkish context.

## Conclusion

The current study translated and adapted the 4Ns Scale by Piazza et al. [42] and the Meat-eating Justifications Scale (MEJ) to Turkish. After specific changes, both measures showed high reliability and validity of several types. Thus, this study will enable future attempts to study meat-eating and meat-eating justifications in Türkiye, which also means enabling cross-cultural studies. Correlates of meat-eating justifications showed that participants who are men, who have higher religious orientation, who are older, who are not actively students, who do not have a graduate education, who have higher social dominance orientation, who have more speciesism, who are more committed to meat, and who attribute less mind to farm animals endorse meat-eating justifications more. In addition, participants who are men, who are undergraduate students, who are younger, who endorse meat-eating justifications more, who have higher social dominance orientation, and who have more speciesism consume meat more frequently. This bundle of information and the adapted measures could be utilized in future attempts to study and reduce meat eating.

## Data Availability

The dataset collected and analyzed for the current study can be accessed here (the link is anonymized; thus, will not reveal the identities of the researchers): https://osf.io/kw3nh/?view_only=a77ecade7cdd4c85908e0b00a08c7210.

## Appendix A

### Final version of the Turkish adaptation of the 4Ns of Meat-Eating Justifications Scale (With English items)

#### Normal (Normal)

1. Et yemek doğal bir davranıştır. (It is natural to eat meat.)
2. Toplumda, et yemenin kabul görmesi normaldir. (It is normal that meat-eating is socially acceptable)
3. Tanıdığım çoğu kişi pek tabii et yer. (Most people I know, no wonder, eat meat.)
4. Et yemek normal bir davranıştır. (It is normal to eat meat.)

#### Gerekli (Necessary)

5. Tamamen bitkisel beslenmek doğal değildir (It is unnatural to eat an all plant-based diet.)
6. Sağlıklı olabilmek için et yemek gereklidir. (It is necessary to eat meat in order to be healthy.)
7. Tamamen bitkisel beslenerek, ihtiyacımız olan proteini, vitaminleri ve mineralleri alamayız. (You cannot get all the protein, vitamins, and mineral you need on an all plant-based diet.)
8. İnsanların et yemesi gerekir. (Human beings need to eat meat.)
9. Sağlıklı bir beslenme düzeninin, biraz da olsa et içermesi gerekir. (A healthy diet requires at least some meat.)

#### Doğal (Natural)

10. Atalarımız hep et yemişlerdir. (Our human ancestors ate meat all the time.)
11. İnsanların canı doğal olarak et çeker. (Human beings naturally crave meat.)
12. Et yemek yaradılışımızda vardır. (Eating meat is by creation.)

#### Güzel (Nice)

13. Et, yemeklere o kadar lezzet katar ki; yemeğe et koymamak saçma olur. (Meat adds so much flavor to a meal it does not make sense to leave it out.)
14. En lezzetli yemekler, tabii ki, et yemekleridir (örn: biftek, ızgara tavuk ve balık). (The best tasting food is normally a meat based dish (e.g., steak, chicken breast, grilled fish)).
15. Etsiz yemekler tatsız tuzsuz olur. (Meals without meat would just be bland and boring.)
16. Et, bazı sebze ve bakliyat yemeklerini yenilebilir hale getirir. (örn: etli kuru fasulye, kıymalı karnabahar). (Meat makes certain vegetable and legume dishes edible (e.g., beans with meat, cauliflower with minced meat).)

## Appendix B

### Final version of the Turkish adaptation of the Meat-Eating Justifications Scale (MEJ) (With English items)

#### Et taraftarlığı (PRO MEAT)

1. Et yemeyi o kadar çok seviyorum ki, ondan asla vazgeçmem. (I enjoy eating meat too much to ever give it up.)
2. Etin tadı o kadar güzeldir ki; et tüketimi ile ilgili yapılan eleştirileri dert etmeye değmez. (Meat tastes too good to worry about what all the critics say.)
3. Lezzetli bir et kadar beni tatmin eden bir yiyecek daha yoktur. (There is no food that satisfies me as much as a delicious piece of meat).

#### Acı çekildiğinin inkarı (DENY)

4. Hayvanlar, etleri için yetiştirilip öldürülürken aslında acı çekmezler. (Animals don’t really suffer when being raised and killed for meat.)
5. Hayvanlar, insanlarla aynı şekilde acı hissetmezler. (Animals do not feel pain the same way humans do.)
6. Et endüstrisi, hayvanın duyduğu acı ve rahatsızlığı en aza indirecek ve hatta engelleyecek şekilde çalışır. (Meat is processed so that animal pain and discomfort is minimized and avoided.)

#### Hiyerarşik Gerekçelendirmeler (HIER. JUST)

7. Bazı hayvanların etini yemek kabul edilebilir çünkü bu hayvanlar yiyelim diye yetiştirilmektedir. (It’s acceptable to eat certain animals because they’re bred for that purpose.)
8. İnsanlar besin zincirinin en üstünde yer aldıkları için hayvanları yemek doğalarında vardır. (Humans are at the top of the food chain and meant to eat animals.)
9. Sonuçta, hayvanlar ihtiyaçlarımızı karşılamak için vardır. (Ultimately, animals are here to serve our needs.)

#### Kaynaktan ayrıştırma (DISSOC.)

10. Et gördüğümde, o etin kaynağı olan hayvanı düşünmemek için özel bir çaba sarf ederim. (When I look at meat, I try hard not to connect it with an animal.)
11. Yediğim etin nereden geldiğini düşünmekten hoşlanmam. (I do not like to think about where the meat I eat comes from.)
12. Et yerken, yediğim hayvanın yaşamını düşünmemeye çalışırım. (When I eat meat, I try not to think about the life of the animal I am eating.)

#### Dini gerekçelendirmeler (REL. JUST)

13. Allah hayvanları yememiz için yaratmıştır. (God intended for us to eat animals.)
14. Allah bize hayvanlara hükmetme hakkı vermiştir. (God gave us dominion over animals.)
15. İnsanların et yemesi Allah’ın emridir. (It is God’s will that humans eat animals.) 

#### Kaçınma (AVOID)

16. Mezbahalarda neler olup bittiğini düşünmemeye çalışırım. (I try not to think about what goes on in slaughterhouses.)
17. Bir mezbahayı gezmek beni rahatsız eder. (I would have problems touring a slaughterhouse.)
18. Konu, yediğimiz hayvanların nasıl acı çektiğini gözümde canlandıracak detaylara getirildiğinde, o konuşmadan uzak durmaya çalışırım. (I try to stay away when people start talking to me in graphic terms about how the animals we eat suffer.)

#### Sağlıkla ilgili gerekçelendirmeler (HEALTH JUST)

19. Güçlü kaslar için et yemek gereklidir. (Meat is essential for strong muscles.)
20. Sağlıklı gelişim için, sadece etten elde edebileceğimiz proteine ihtiyacımız vardır. (We need the protein we can only get in meat for healthy development.)
21. Sağlıklı beslenmek için ete ihtiyacımız vardır. (We need meat for a healthy diet.)

#### İnsan doğasıyla ilgili gerekçelendirmeler (HD/FATE JUST)

22. Bilim insanlarının, vücudumuzun et yemeyi kolaylaştıracak şekilde evrildiğine inandığını öğrenmek beni şaşırtmaz (örn: dişlerimizin şu anki haline evrilmesi). (It wouldn’t surprise me to learn that scientists believe the human body (e.g., our teeth) has evolved to eat meat.)
23. Et yemeyi bırakmak, evrime ve insanın fıtratına aykırıdır. (It violates human destiny and evolution to give up eating meat.)
24. Tıpkı atalarımız gibi, bizim de et yememiz gerekir. (Our early ancestors ate meat, and we are supposed to also.)

## Abbreviations

4Ns: Natural, Necessary, Normal, Nice
AS: Ambivalent Speciesism Scale
CFA: Confirmatory Factor Analysis
CFI: Comparative Fit Index
CI: Confidence Interval
DSCF: Dwass–Steel–Critchlow–Fligner test
EFA: Exploratory Factor Analysis
MAS: Mind Attribution Scale
MEJ: Meat‑Eating Justification Scale
MCS: Meat Commitment Scale
MRNS: Male Role Norms Scale
RMSEA: Root Mean Square Error of Approximation
SDO: Social Dominance Orientation
SDO-D: Social Dominance Orientation – Dominance
SDO-E: Social Dominance Orientation – Anti‑egalitarianism
SRMR: Standardized Root Mean Square Residual
χ²/df: Chi‑square to degrees‑of‑freedom ratio
α: Cronbach’s alpha
ω: McDonald’s omega
